# Insight into antioxidant-like activity and computational exploration of identified bioactive compounds in *Talinum triangulare* (Jacq.) aqueous extract as potential cholinesterase inhibitors

**DOI:** 10.1186/s12906-024-04424-2

**Published:** 2024-03-28

**Authors:** Olakunle Bamikole Afolabi, Oluwaseun Ruth Olasehinde, Olutunmise Victoria Owolabi, Kikelomo Folake Jaiyesimi, Funmilayo Deborah Adewumi, Olajumoke Tolulope Idowu, Samson Olatunde Mabayoje, Adejoke Olukayode Obajuluwa, Oghenerobor Benjamin Akpor

**Affiliations:** 1 Department of Chemical Sciences, Phytomedicine and Toxicology Unit, College of Sciences, Biochemistry ProgrammeAfe-Babalola University, P.M.B 5454, Ado- Ekiti, Ekiti State Nigeria; 2 Department of Medical Biochemistry, College of Medicine and Health Sciences, Afe Babalola University, P.M.B 5454, Ado-Ekiti, Ekiti State Nigeria; 3 Department of Chemical Sciences, Industrial and Chemical Unit, Industrial Chemistry Programme, College of Sciences, Afe-Babalola University, P.M.B 5454, Ado- Ekiti, Ekiti State Nigeria; 4 Department of Biological Sciences, College of Sciences, Afe-Babalola University, P.M.B 5454, Ado- Ekiti, Ekiti State Nigeria

**Keywords:** Cholinesterase inhibitors, *Talinum triangulare*, Phytonutrients, Alzheimer’s disease, Drug development

## Abstract

**Background:**

Recent reports have highlighted the significance of plant bioactive components in drug development targeting neurodegenerative disorders such as Alzheimer’s disease (AD). Thus, the current study assessed antioxidant activity and enzyme inhibitory activity of the aqueous extract of *Talinum triangulare* leave (AETt) as well as molecular docking/simulation of the identified phytonutrients against human cholinesterase activities.

**Methods:**

In vitro assays were carried out to assess the 2,2- azinobis (3-ethyl-benzothiazoline-6-sulfonic acid) (ABTS) cation radicals and cholinesterase inhibitory activities of AETt using standard protocols. High performance liquid chromatography coupled with diode-array detection (HPLC–DAD) was employed to identify compounds in AETt. Also, for computational analysis, identified bioactive compounds from AETt were docked using Schrodinger's GLIDE against human cholinesterase obtained from the protein data bank (https://www.rcsb.org/).

**Results:**

The results revealed that AETt exhibited a significant concentration-dependent inhibition against ABTS cation radicals (IC50 = 308.26 ± 4.36 µg/ml) with butylated hydroxytoluene (BHT) as the reference. Similarly, AETt demonstrated a significant inhibition against acetylcholinesterase (AChE, IC50 = 326.49 ± 2.01 µg/ml) and butyrylcholinesterase (BChE, IC50 = 219.86 ± 4.13 µg/ml) activities with galanthamine as the control. Molecular docking and simulation analyses revealed rutin and quercetin as potential hits from AETt, having showed strong binding energies for both the AChE and BChE. In addition, these findings were substantiated by analyses, including radius of gyration, root mean square fluctuation, root mean square deviation, as well as mode similarity and principal component analyses.

**Conclusion:**

Overall, this study offers valuable insights into the interactions and dynamics of protein–ligand complexes, offering a basis for further drug development targeting these proteins in AD.

**Supplementary Information:**

The online version contains supplementary material available at 10.1186/s12906-024-04424-2.

## Introduction

Alzheimer’s disease (AD) is a prevalent age-related neurodegenerative disease and a leading cause of dementia [1]. It is characterized by progressive memory loss and cognitive decline, due to a persistent neurodegeneration and brain atrophy [2, 3]. The prevalence of AD has been reported to range from 3% in individuals aged 65 years to 47% in those aged 85 years and older, affecting approximately 15 million people worldwide [4–6]. This disease has become a severe medical problem in modern society as the population increases [7]. Significantly, while fatalities from cardiovascular diseases and prostate cancer decreased between 2000 and 2015, there was a notable increase in deaths associated with AD [5, 8]. Studies have unveiled a decline in cholinergic neurons, resulting in both structural and functional impairment in AD [9, 10]. Likewise, there are signs indicating that the pathogenesis and advancement of AD are markedly affected by heightened oxidative stress and cholinergic dysfunction [11].

Acetylcholinesterases, AChE (EC 3.1.1.7) and butylrycholinesterase, BChE (EC 3.1.1.8), are a group of serine hydrolases responsible for the hydrolysis of acetylcholine (ACh) and butyrylcholine (BCh) into choline and acetic acid or butyric acid [12, 13]. The hydrolytic actions of these cholinesterases (ChE) have been reported to alter the levels of the cholinergic system, resulting into imbalance or disturbance in cholinergic signaling systems essential for proper neurotransmission and cognitive functions [14]. Both enzymes exhibit approximately 65% structural homology, and BChE primarily assumes a supportive role and contributes to approximately 10% of the overall ChE activities, specifically within the temporal cortex [15]. Inhibition of the enzymatic activities of AChE and BChE are particularly significant in sustaining acetylcholine and its activity at cholinergic synapses for normal cognitive function in AD and other dementia disorders [13, 16].

Presently, investigations toward discovering new target drugs capable of preventing both the pathophysiogenesis and progression of numerous human diseases are on going in the field of science [9]. The therapeutic approaches that involve inhibition of cholinesterase and proliferation of ROS/RNS have been reported as crucial measures in the management of AD [17]. Several AChE inhibitors including rivastigmine, galantamine, donepezil, memantine, among others are readily available and in use for clinical attention in AD condition [18]. These chemotherapeutic agents are symptomatic with a temporary relief from AD by elevating Ach level in the brain [19]. Recently, naturally-derived bioactive compounds from plants are widely being exploited for their varied range of biological interactions and activities [20, 21]. These compounds possess the ability to scavenge and inhibit the production of reactive species via their tendency to donate electron and stabilize these electrophiles [22].

*Talinum triangulare* (*Tt*), waterleaf is a dicotyledonous plant belonging to the family *Talinaceae* and commonly grown in humid tropics with alternate, simple, and succulent leaves [23]. The plant is commonly utilized in the preparation of soups and other culinary delicacies [24]. Research studies have revealed that the plant is rich in natural compounds like flavonoids and polyphenols [25, 26]. The plant has exhibited a wide range of biological and pharmaceutical properties, including anti-inflammatory, anti-fungal, neuroprotective, and anti-bacterial activities [27–29]. Additionally, in our previous studies, we reported the antioxidant properties [30], and presence of a number of bioactive compounds identified from the aqueous extract of *Tt* [25]. However, there is a paucity of information on computational interactions of these compounds with AChE and BChE activities in the prevention/management of neurodegeneration diseases such as AD. Hence, this study aimed to explore the potential cholinesterase inhibitory activities of aqueous extract of *Tt* leave (AETt) as well as investigating computer-aided interactions of available bioactive components for possible drug-like hits using in vitro and insilico approaches.

## Materials and methods

### Chemicals and reagents used

The chemicals and reagents which include acetylcholine iodide, butyrylcholine iodide, galanthamine (galantamine hydrobromide Reminyl ®), 2,2- azinobis (3-ethyl-benzothiazoline-6-sulfonic acid) (ABTS), butylated hydroxytoluene (BHT) Ellman’s reagent (5,5’-dithiobis (2-nitrobenzoic acid), DTNB) were procured from Sigma-Aldrich, Inc., (Saint Louis, MO). Other reagents used in this experiment were of analytical grade and prepared using sterilized distilled water in all-glass apparatus.

### Plant collection and processing

Fresh ariel leaves of *Tt* were purchased from the popular King’s market in Ado-Ekiti, Ekiti State, Nigeria. A voucher sample was subsequently taken to the Department of Plant Science at Ekiti State University, Ado-Ekiti, Ekiti State, Nigeria, for authentication and identification. The sample was identified by a taxonomist in the department and assigned Herbarium number UHAE 2013/76, following thorough taxonomic investigations from the database.

### Preparation of aqueous extract of *Talinum triangulare*

The leaves were properly washed and air-dried at room temperature (RT, 25 °C) for two weeks to obtain a constant weight and pulverized to powdery form using automated blender. A quantity of 50 g of the powdered sample was extracted with 500 ml of distilled water for a period of 48 h and concentrated at 55 °C using water-bath to achieve AETt. Threafter, different concentrations were prepared from a stock solution obtained from the resulting extract and then subjected to different bioassays.

### Antioxidant activity of AETt using ABTS inhibitory assay

The ABTS cation radical scavenging ability assay of AETt was carried out according to the method described of Miller et al. [31] with minor modifications. A volume of 0.2 ml of the sample at various concentrations was mixed with 2.0 ml of a diluted ABTS radical cation solution (7 mM ABTS dissolved in 0.01 M PBS at pH 7.4). The reaction mixture was then allowed to stand at RT for 20 min, and the absorbance was immediately measured at 734 nm using a UV spectrophotometer. The ABTS free radical scavenging ability of AETt was calculated and expressed as percentage (%) inhibition with BHT as standard control using:

$$ABTS\;cation\;scavenging\;ability\;(\%)\:=\:(Abs_{control}\;-\;Abs_{sample})/\;Abs_{control}\:\times\:100$$

Where; Abs _control_ = Absorbance of the reaction mixture in absence of the extract (control).

Abs _sample_ = Absorbance of the reaction mixture (in presence of the extract).

### Cholinesterase inhibitory activity assay

Cholinesterase (AChE and BChE) inhibitory activity assay of the AETt was performed using a colorimetric method as described by Ellman et al. [32]. The AChE/ BChE activity was determined in a reaction mixture with total volume of 1 ml, comprising 0.1 M phosphate buffer (pH 8.0), 10 mM DTNB, 0.05 ml cytosol, and 150 mM acetylcholine iodide or butyrylcholine iodide in the presence/absence of the inhibitor (different concentrations of the extract and control). The reaction mixture was monitored for a change in absorbance at 412 nm using a UV spectrophotometer at RT for a duration of 3 min. Following the analysis, the inhibitory activity of the extract against AChE or BChE was calculated and expressed as a percentage inhibition of the control as follows:

$$AChE/BChE\;inhibitory\;activity\;(\%)\:=\:(Abs_{control}\;-\;Abs_{sample})/\;Abs_{control}\:\times\:100.$$

Where; Abs _control_ = Absorbance of the reaction mixture without the extract (control).

Abs _sample_ = Absorbance of the reaction mixture with the extract.

### Determination of IC_50_ values

The IC_50_ value (µg/ml), representing the concentration of AETt required to cause 50% inhibition was determined through the utilization of a linear regression curve generated with Microsoft® excel 2016 as described by Afolabi et al. [9]. This curve was generated by plotting the percentage inhibition caused by the extract against different concentrations (µg/ml) of the extract used. The straight line equation (y = mx + c) derived from the curve plotted was used to determine the values, where y represented % inhibition at 50%; m, slope; x, concentration that caused 50% inhibition; and c, intercept.

### In silico and molecular simulation studies

#### Preparation of protein targets and ligands

The X-ray crystal structures of human acetylcholinesterase (AChE) (PDB ID: 4EY7) and human butyrylcholinesterase (BChE) (PDB ID: 6QAE) were acquired from the Protein Data Bank (https://www.rcsb.org/). Subsequently, the obtained structures were subjected to further preparation using the protein preparation wizard feature in Glide. Additionally, all compounds obtained from the HPLC–DAD analysis, as well as the standard drugs for individual targets (obtained from MedExpress and DrugBank), were prepared using LigPrep 2.4 software as reported by Mahmoud et al. [33]. The optimization process utilized the OPLS-2005 force field, which led to the generation of low-energy conformers for each ligand [34].

#### Molecular docking modelling using Maestro

The HPLC–DAD identified compound from the AETt, along with known inhibitors (drugs), were subjected to molecular docking into the AChE (PDB ID: 4EY7) and BChE (PDB ID: 6QAE) using Schrodinger's grid-based ligand docking with energetics (GLIDE) software version 5.8, following the method described by Halgren [35]. For docking ligands, the Glide 5.6 software's receptor grid generation module (GRGGM) was utilized to define the active sites. Grids were generated around the active sites of 4EY7 and 6QAE using receptors with a van der Waals scale of 0.9 for non-polar atoms, and co-crystallized ligands were used as references. Two distinct docking techniques were employed: standard precision (SP) and high precision (XP) to explore the binding modes of the compounds and known inhibitors for each target as described by Friesner [36].

#### Prime MM/GBSA calculation

The Prime/MM-GB/SA technique was employed to calculate the free energy of binding for a specific set of ligands to a receptor using the OPLS-AA force field and the generalized-Born/surface area (GB/SA) continuum solvent model as described below [37].

1 $$\Delta {{\text{G}}}_{\mathrm{binding }}=\mathrm{ \Delta E}+\Delta {{\text{G}}}_{{\text{solvation}}}+\Delta {{\text{G}}}_{{\text{SA}}}$$

2 $$\mathrm{\Delta E }= {{\text{E}}}_{{\text{complex}}}- {{\text{E}}}_{{\text{protein}}}-{{\text{E}}}_{{\text{ligand}}}$$

Where in (1 & 2) ΔG_binding_, binding free energy; ΔG_SA,_ free energy of surface area; ΔG_solvation_^.^ solvation free energy (1); ΔE, free minimized energy; E_complex_, E_protein_, and E_ligand_ are the minimized energies of the protein–inhibitor complex, protein and inhibitor, respectively.

3 $$\Delta {{\text{G}}}_{{\text{solvation}}} = {{\text{G}}}_{\mathrm{solvation }({\text{complex}})}-{{\text{G}}}_{\mathrm{solvation }\left({\text{protein}}\right)}-{{\text{G}}}_{\mathrm{solvation }({\text{ligand}})}$$

Where in (3); G_solvation (complex)_, G_solvation (protein)_, and G_solvation (ligand)_ are the solvation free energies of the complex, protein, and inhibitor (ligand), respectively:

4 $$\Delta {{\text{G}}}_{{\text{SA}}} = {{\text{G}}}_{\mathrm{SA }({\text{complex}})}-{{\text{G}}}_{\mathrm{SA }\left({\text{protein}}\right)}-{{\text{G}}}_{\mathrm{SA }({\text{ligand}})}$$

Where in (4); G_SA (complex)_, G_SA (protein)_, and G_SA (ligand)_ are the complex, protein, and inhibitor surface area energies, respectively.

#### ADMET/ADME and druglikeness analysis

The SwissADME web tool and ADMETlab 2.0 were employed to predict the ADMET, druglikeness, and medicinal chemistry parameters of rutin and quercetin as described by Daina et al. [38]. The tool is accessible at http://www.swissadme.ch/.

#### Molecular dynamics simulation and RMSD analyses

The protein–ligand complexes of rutin and quercetin as obtained from the molecular docking step were subjected to molecular dynamics simulation (MDS) over 20 nano seconds (ns) using GROMACS software 2018 [39], in order to understand the effect of their binding on the structural stability and conformational flexibility of protein–ligand complexes.

#### Statistical analyses

Data were analyzed using GraphPad Prism 8.0 (Version 8, Software Program, GraphPad Prism Inc., San Diego, CA). Results were presented as mean ± SD. One-way analysis of variance (ANOVA) was used for the analyses, followed by Tukey’s post-hoc test. Significant differences were considered at *p* < 0.05.

## Results

### Antioxidant property and cholinesterase inhibitory activities of the aqueous extract of *Talinum triangulare* (AETt) leave

**Fig. 1:**
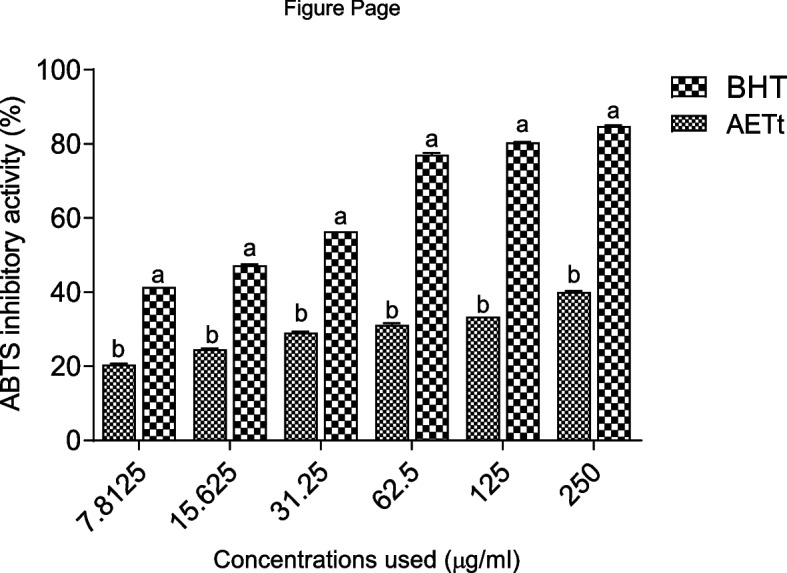
ABTS inhibitory activity of the aqueous extract of *Talinum triangulare* leave. Key: **a** & **b** represent levels of significance at *p* < 0.05 among different concentrations used for the samples, i.e., across bars; *BHT* butylated hydroxytoluene, *AETt* aqueous extract of *Talinum triangulare* leave

Figure 1 shows the inhibitory activity of AETt against ABTS free cation radical. As shown in the result, AETt demonstrated a significant (p < 0.05) inhibition in a concentration-dependent manner against ABTS cation radical (IC_50_ = 308.26 ± 4.36 µg/ml) compared to BHT (IC_50_ = 48.23 ± 0.18 µg/ml) (Table 1).

**Table 1:** IC_50_ (µg/ml) of the inhibitory activities of AETt against ABTS, BchE and AchE

	AETt	Galanthamine	BHT
ABTS	308.26 ± 4.36	-	48.23 ± 0.18
AchE	326.49 ± 2.01	57.36 ± 0.04	-
BchE	219.86 ± 4.13	51.37 ± 0.28	-

Results represent mean ± SD of duplicate trials (*n* = 2)

**Fig. 2:**
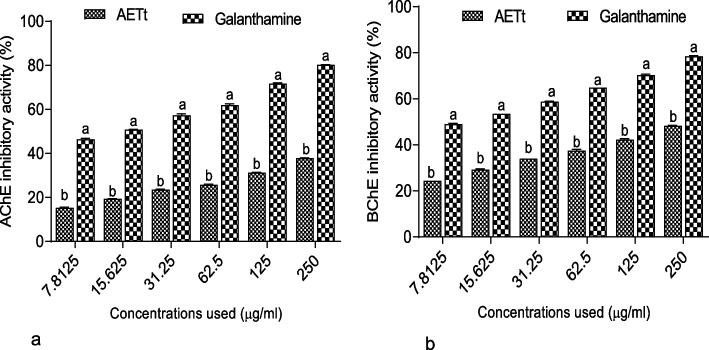
Inhibitory activity of the aqueous extract of *Talinum triangulare* leave against **a** AChE and **b** BChE activities*.* Key: **a** & **b** represent levels of significance at *p* < 0.05 among different concentrations used for the samples, i.e., across bars; *AETt* aqueous extract of *Talinum triangulare* leave

Figure 2 a&b reveal inhibitory activities of the AETt against AChE and BChE activities. As shown in the results, AETt revealed a significant (*p* < 0.05) inhibition against AChE activity (IC_50_ = 326.49 ± 2.01 µg/ml) compared to galanthamine (IC_50_ = 57.36 ± 0.04 µg/ml) (Table 1), similarly, the extract had a higher inhibition against BChE activity (IC_50_ = 219.86 ± 4.13 µg/ml) compared to galanthamine (IC_50_ = 51.37 ± 0.28 µg/ml) in a concentration-dependent manner.

### Molecular docking analyses

**Fig. 3:**
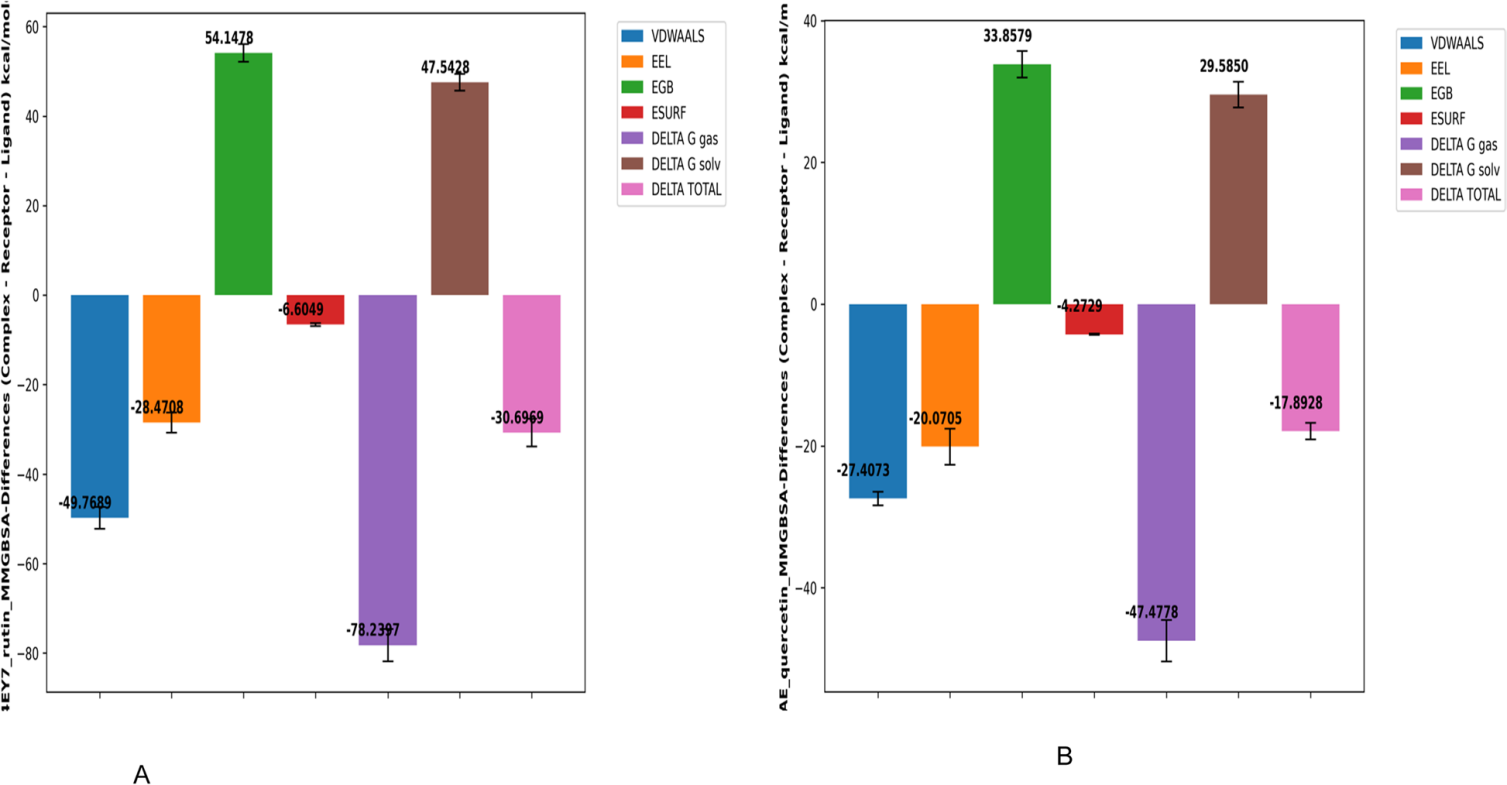
**a**&**b** Molecular mechanics/generalized born surface area value (MMGBSA) for **a** rutin dynamics binding to AChE; **b** quercetin dynamics binding to BChE. Note: *VDWAAL* Van der Waals, *EEL* Electrostatic, *EGB* Generalized born electrostatic, *ESURF* Nonpolar solvation, *DELTA G gas* Calculated free energy of binding (ΔG gas), *DELTA G Solv* Solvation contribution, *DELTA TOTAL* overall binding energy (ΔG total)

Figure 3 (a&b) represent the molecular mechanics/generalized born surface area value (MMGBSA) for (a) rutin dynamics binding to AChE; (b) quercetin dynamics binding to BChE. As indicated in Fig. 3a, MMGBSA analysis for rutin binding to AChE revealed energy components, including Van der Waals (VDWAALS) at -49.77 ± 2.45 kcal/mol, electrostatic (EEL) at -28.47 ± 2.27 kcal/mol, generalized born electrostatic (EGB) at 54.15 ± 1.96 kcal/mol, and nonpolar solvation (ESURF) at -6.60 ± 0.35 kcal/mol. The calculated free energy of binding (ΔG gas) was -78.24 ± 3.59 kcal/mol, with a significant solvation contribution (ΔG solv) of 47.54 ± 1.87 kcal/mol. The overall binding energy (ΔG total) was -30.70 ± 3.05 kcal/mol. Similarly, for quercetin binding to BChE (Fig. 3b), the MMGBSA analysis yielded energy components, including VDWAALS at -27.41 ± 0.95 kcal/mol, EEL at -20.07 ± 2.55 kcal/mol, EGB at 33.86 ± 1.88 kcal/mol, and ESURF at -4.27 ± 0.09 kcal/mol. The calculated ΔG gas was -47.48 ± 2.93 kcal/mol, with a ΔG solv of 29.59 ± 1.81 kcal/mol. The overall ΔG total was -17.89 ± 1.19 kcal/mol.

**Fig. 4:**
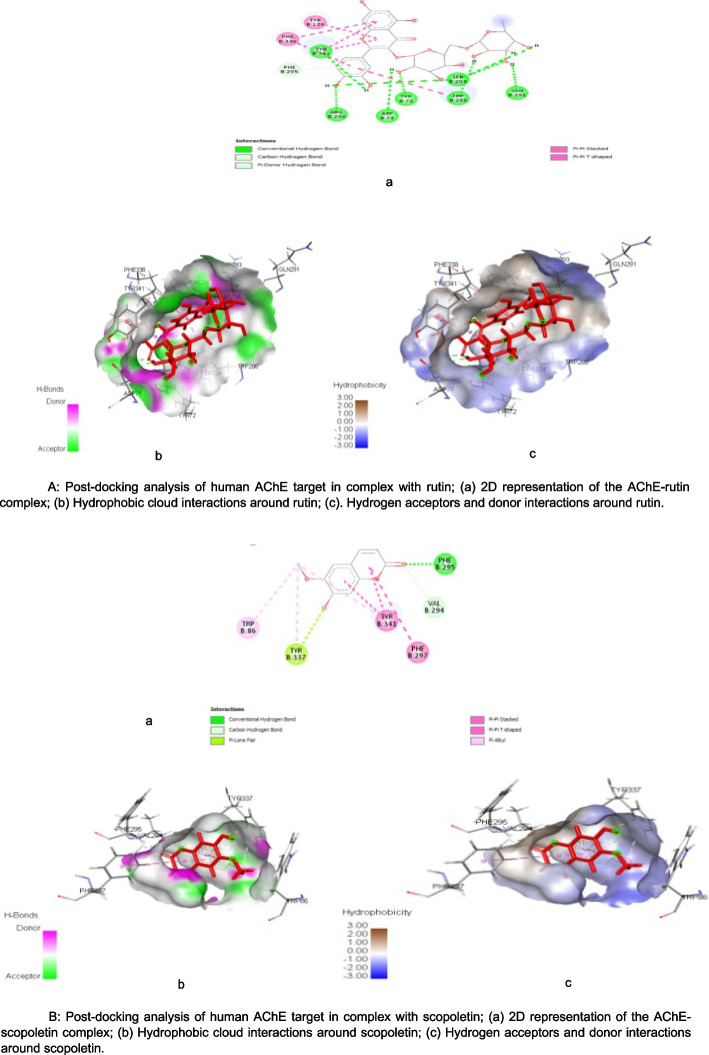
**A** Post-docking analysis of human AChE target in complex with rutin; a 2D representation of the AChE-rutin complex; b Hydrophobic cloud interactions around rutin; c. Hydrogen acceptors and donor interactions around rutin. **B** Post-docking analysis of human AChE target in complex with scopoletin; a 2D representation of the AChE-scopoletin complex; b Hydrophobic cloud interactions around scopoletin; **c** Hydrogen acceptors and donor interactions around scopoletin

Figure 4 (A & B) show post-docking analyses of human AChE protein target in complex with rutin and scopoletin (reference drug). The 2D representation of the human AChE-rutin complex shown in the Fig. 4A (a) indicated SP (-14.34 kcal/mol), XpGscore (-14.37 kcal/mol) and MM-GBSA dG bind score of -73.21 kcal/mol. However, in the Fig. 4B (a) scopoletin (reference drug) in complex with the same targets revealed SP (-7.91 kcal/mol), XpGscore (-7.92 kcal/mol) and MM-GBSA dG Bind score of -37.87 kcal/mol, respectively (Table 2). Also, as shown in Fig. 4A (b), the interaction of rutin (hit) with this target showed twelve hydrogen bond formation at specific bond distances of TYR72 (1.7046), SER293 (2.43506), SER293 (1.75982), ARG296 (2.23347), ASP74 (2.80258), TRP286 (1.95637), GLN291 (2.24675), SER293(1.90847), TYR341 (1.96344), SER293 (2.72111), SER293 (2.19421), and PHE295 (3.22904), in addition to seven hydrophobic bond (Fig. 4A (c)) which included TYR341 (3.74547), TYR341 (3.80462), TYR124 (5.50253),TRP286 (4.86732), PHE338 (5.053), and PHE338 (5.53433), which are precisely alkyl, Pi-Pi T-shaped, and Pi-Pi Stacked. However, in contrast to the interactions formed by rutin with the same target, scopoletin interaction with the human AChE (Fig. 4B (b)) revealed the formation of three hydrogen bond interactions at specific bond distances of PHE295 (1.88968), VAL294 (2.70336), and TYR337 (2.95665), in addition to six hydrophobic bond interactions formed at TYR341 (3.93256), TYR341 (3.76354), PHE297 (5.88588), TRP86 (4.83487), TYR337 (5.07224), and TYR341 (5.4545), respectively (Fig. 4B (c)).

**Table 2:** Post docking SP, XPG and MMGBSA scores of compounds from the AETt against human acetylcholinesterase target with scopoletin as standard

PubMed IUPAC	Docking score	XPG score	MMGBSA dG binding score
Rutin	-14.34	-14.37	-73.21
Quercetin	-12.16	-12.19	-59.43
Luteolin	-11.71	-11.75	-57.76
Kaempferol	-10.69	-10.72	-45.85
Scopoletin	-7.91	-7.92	-37.87

**Fig. 5:**
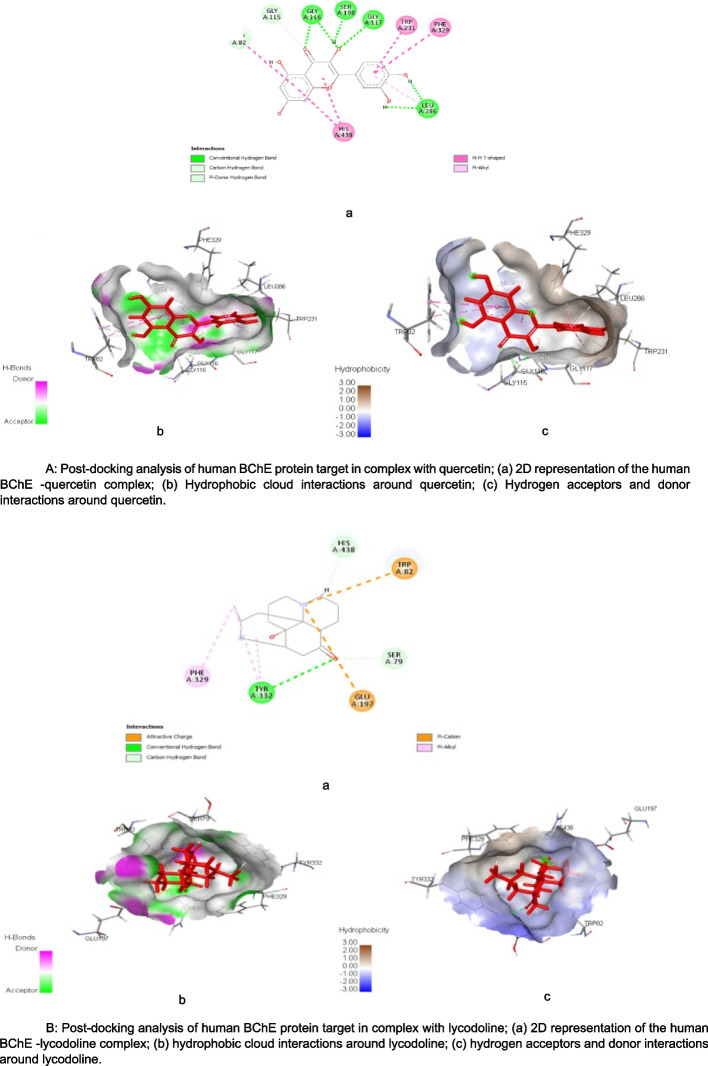
**A** Post-docking analysis of human BChE protein target in complex with quercetin; **a** 2D representation of the human BChE -quercetin complex; **b** Hydrophobic cloud interactions around quercetin; **c** Hydrogen acceptors and donor interactions around quercetin. **B** Post-docking analysis of human BChE protein target in complex with lycodoline; **a** 2D representation of the human BChE -lycodoline complex; **b** hydrophobic cloud interactions around lycodoline; **c** hydrogen acceptors and donor interactions around lycodoline

Figure 5 (A & B) show the post-docking analyses of human but BChE protein target in complex with quercetin and lycodoline (reference drug). The binding of human BChE protein target to quercetin as shown in Fig. 5A (a) revealed SP (-10.44 kcal/mol), XpGscore (-10.47 kcal/mol) and MM-GBSA dG bind score of -48.36 kcal/mol (Table 3). In contrast, lycodoline interaction with the same target as shown in Fig. 5B (a), indicated SP (-7.35 kcal/mol), XpGscore (-7.53 kcal/mol) and MM-GBSA dG bind score of -50.37 kcal/mol. Similarly, as shown in Fig. 5A (b) the interaction of quercetin with the human BChE revealed the formation of nine hydrogen bond interactions at specific bond distances of GLY116 (1.89663), GLY116 (2.46613), GLY117 (1.90848), SER198 (1.65067), LEU286 (1.9874), LEU286 (1.623), GLY115(2.63459), TRP82 (3.13666), and TRP82 (2.45968), in addition to the eight hydrophobic interactions formed at TRP82 (4.87994), TRP82 (5.34937), TRP231 (5.07094), TRP231 (4.88867), PHE329 (5.0448), HIS438 (4.51515), HIS438 (5.03233), LEU286 (5.09586) (Fig. 5A (c)). In contrast to the interactions formed with quercetin, interaction of lycodoline with human BChE (Fig. 5B (b)) revealed three hydrogen bond interactions formed at specific bond distances of GLU197 (5.15888), TYR332 (2.85079), and SER79 (2.40171), in addition to five hydrophobic interactions formed at HIS438 (2.44777), TRP82 (4.94182), PHE329 (4.33196), TYR332 (4.91587), and TYR332 (3.59395), respectively (Fig. 5B (c)). These was in contrast to the interactions formed when Quercetin interact with the same target.

**Table 3:** Post docking SP, XPG and MMGBSA scores of compounds from the AETt against human butyrylcholinesterase target with Lycodoline as standard

PubMed IUPAC	Docking score	XPG score	MMGBSA dG binding score
Rutin	-12.92	-12.95	-29.28
Quercetin	-10.44	-10.47	-48.36
Luteolin	-9.98	-10.02	-36.77
Kaempferol	-8.84	-8.87	-39.78
Lycodoline	-7.35	-7.53	-50.37

### Molecular dynamics simulation analyses

**Fig. 6:**
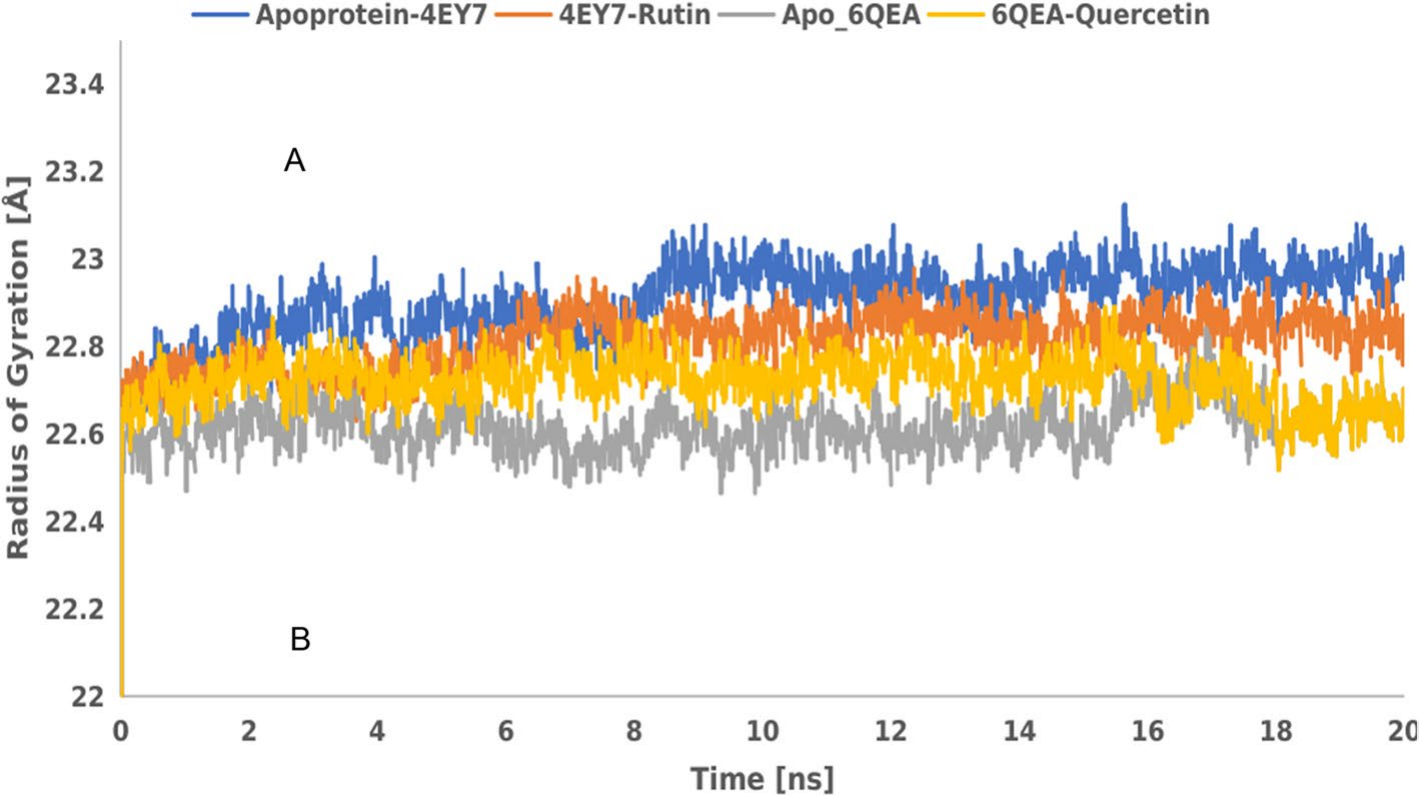
Radius of gyration plot as a function of simulation time-dependent analysis of molecular dynamics trajectory of apo-AChE, AChE-rutin, apo- BChE, and BChE-quercetin complexes

Figure 6 represents radius of gyration (Rg) plot as a function of simulation time-dependent analysis of molecular dynamics trajectory of rutin and quercetin dynamics in complex with AChE and BChE. As indicated in the result, Rg values of the two apoproteins and their protein–ligand complexes in apo-AChE, AChE-rutin complex, apo-BChE and BChE-quercetin complex were 22.88320008, 22.78768594, 22.59427599 and 22.6976769, respectively.

**Fig. 7:**
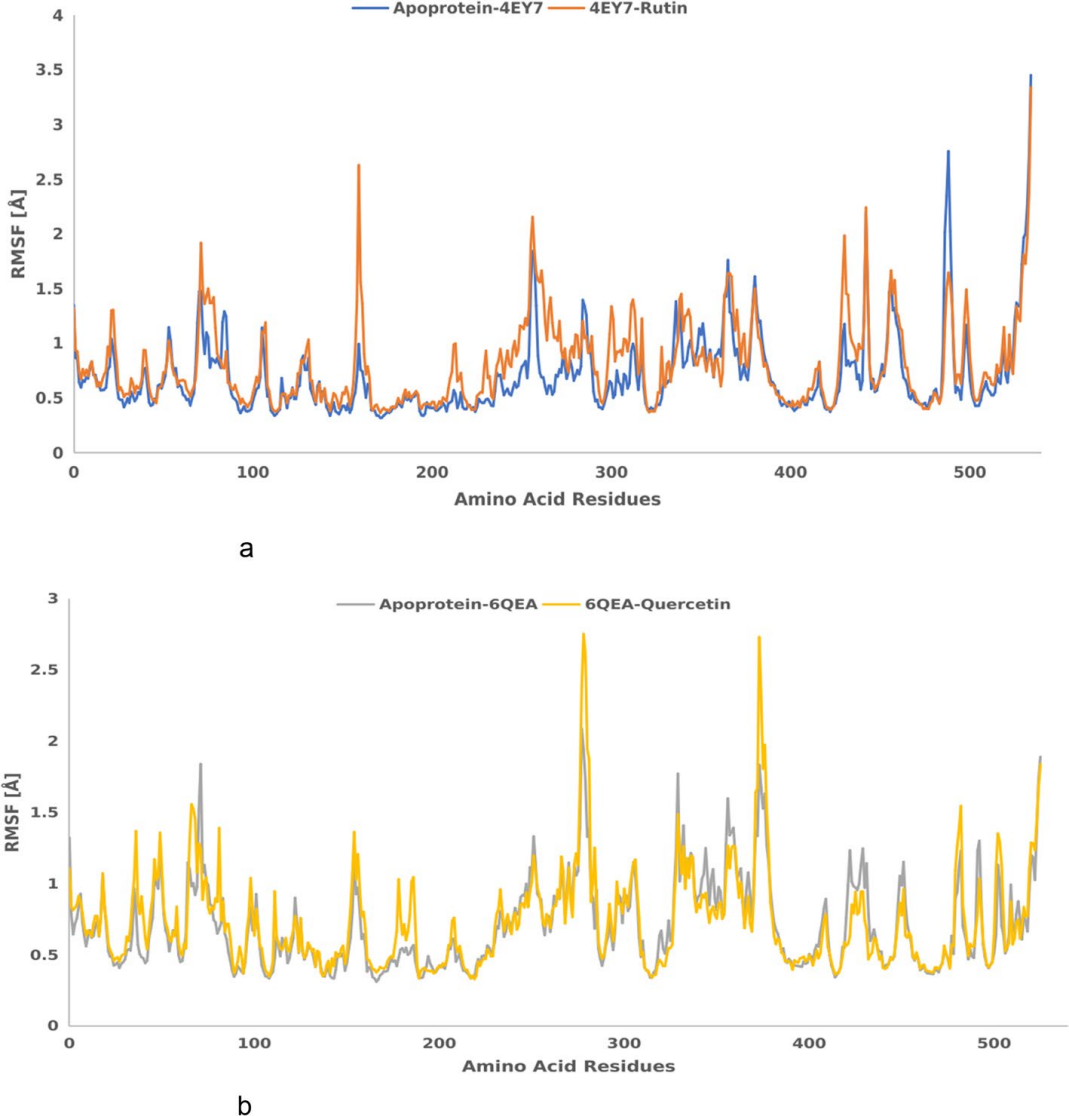
**a**&**b** Root mean square fluctuation of the residues of simulation time dynamics of **a** apo-AChE & AChE-rutin complexes; and **B** apo-BChE & BChE-quercetin complexes

**Fig. 8:**
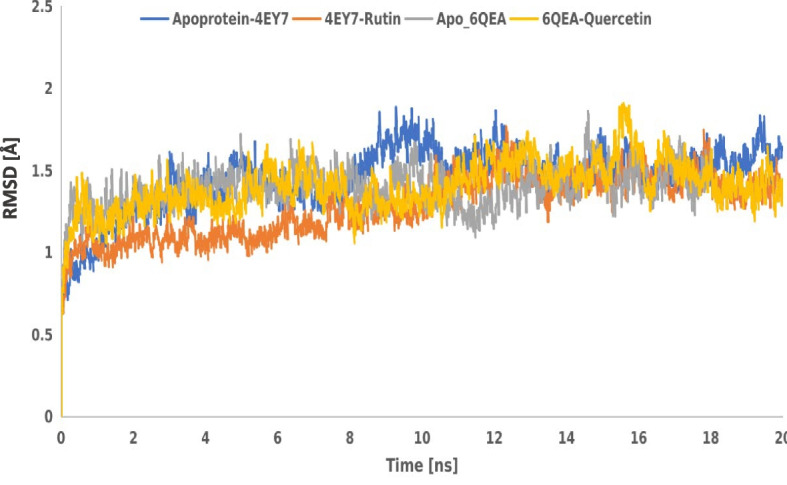
Root mean square deviation (RMSD) plot as a function of simulation time of rutin and quercetin dynamics binding to acetylcholinesterase (PDB ID: 4EY7) and butyrylcholinesterase (PDB ID: 6QAE)

Figure 7 (a&b) represent root mean square fluctuation of the residues of simulation time dynamics of (a) apo-AChE and AChE -rutin complexes; and (b) apo-BChE & BChE-quercetin complexes. As indicated in the plot, RMSF values of the two apoproteins and their protein–ligand complexes were apo-AChE (0.706342291), AChE -rutin (0.810035426), apo-BChE (0.705936449), and BChE-quercetin (0.733836349), respectively. Similarly, Fig. 8 represents the root mean square deviation (RMSD) plot as a function of simulation time of rutin and quercetin dynamics binding to acetylcholinesterase and butyrylcholinesterase. As shown in the plot, the average RMSD values recorded for the apoprotein systems and protein–ligand complexes for apo-acetylcholinesterase, acetylcholinesterase-rutin, apo-butyrylcholinesterase, and butyrylcholinesterase-quercetin complexes were 1.450951857, 1.284406342, 1.391370699, and 1.402250976, respectively.

**Fig. 9:**
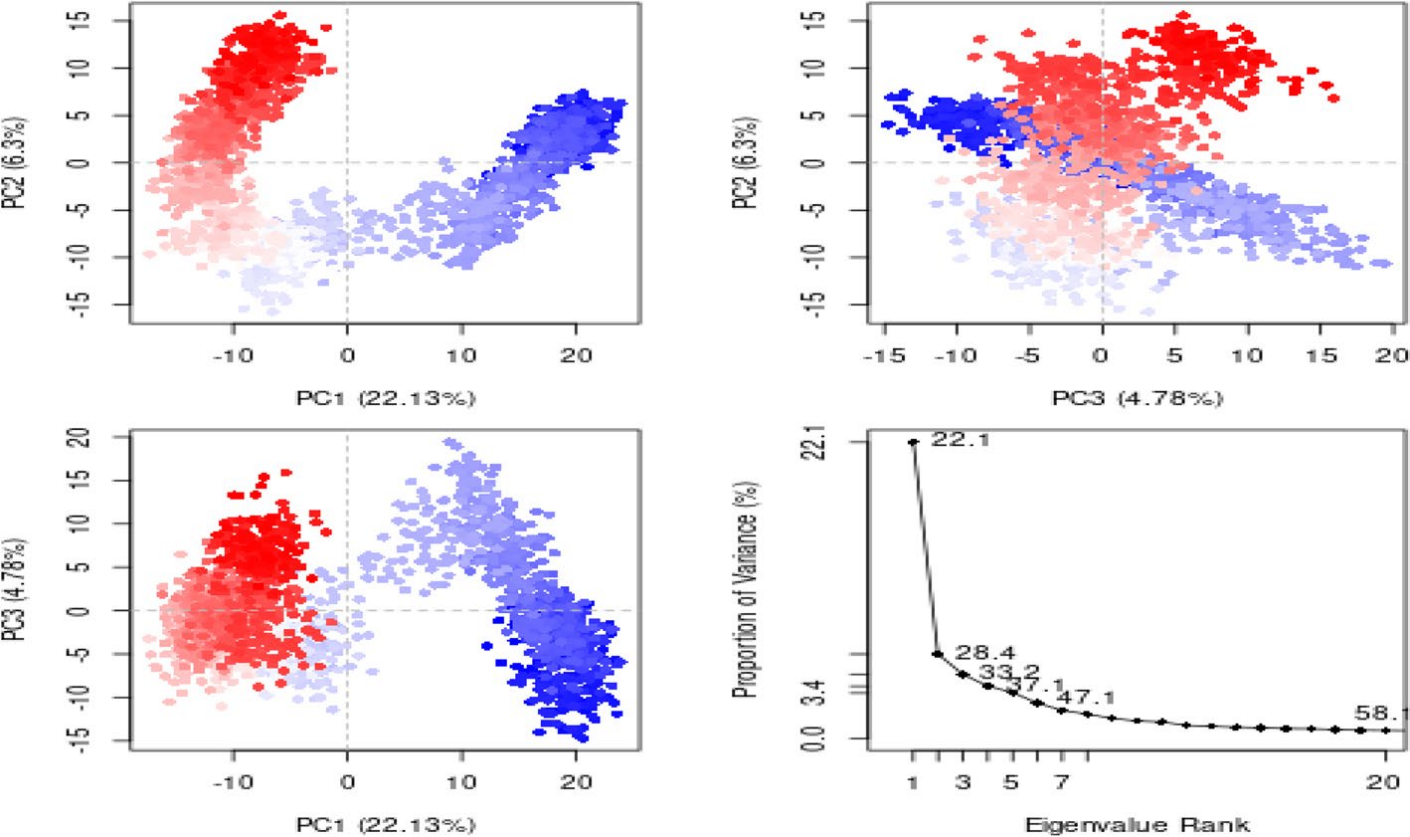
Interpretation of variance of AChE-rutin complex against eigenvalues calculated by principal component (PC) analysis. PCA trajectory with instantaneous conformations (i.e. trajectory frames) colored from blue to red in order of time. The 3 PCs showed fluctuating regions with 33.78% overall fluctuations. The fluctuations in PC1, PC2 and PC3 were 22.1%, 6.3% and 4.78%, respectively

**Fig. 10:**
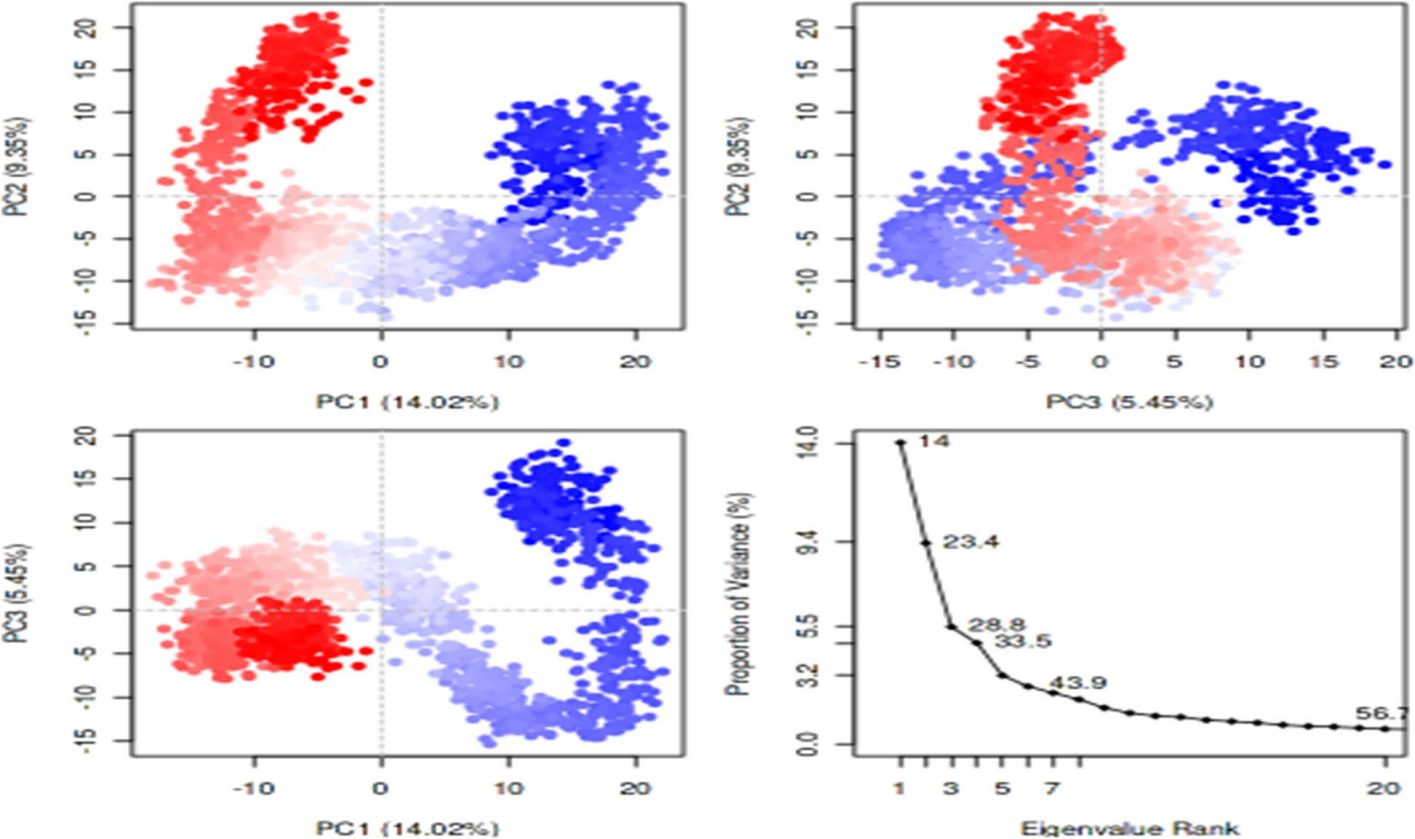
The interpretation of variance of BChE-quercetin complex against eigenvalues calculated by principal component analysis. PCA trajectory with instantaneous conformations (i.e. trajectory frames) colored from blue to red in order of time. The 3 PCs showed fluctuating regions with 33.78% overall fluctuations. The fluctuations in PC1, PC2 and PC3 were 14.02%, 9.35% and 5.45%, respectively

Figures 9 and 10 represent a plot of the receptors’ PC1, PC2, PC3, and eigenvalues versus the corresponding eigenvector indices for the different modes of motion in AChE-rutin and BChE-quercetin complexes. As indicated in Fig. 9, for the AChE-rutin, PC1 had the highest variance with 22.13%, followed by PC2 with 6.3%, and PC3 with 4.78%. Similarly, for the BChE-quercetin (Fig. 10), PC1 had a lower variance of 14.02%, while PC2 and PC3 had higher variances of 9.35% and 5.45%, respectively.

## Discussion

The utilization of medicinal plants has gained a significant attention due to their vital roles in the management of several human ailments [40]. In recent times, this has sparked immense interest in investigating the diverse bioactive compounds involved and mechanisms of their actions [41]. Hence, our study explored in vitro antioxidant and enzyme inhibitory properties as well as interactions of bioactive compounds from AETt with AChE and BChE activities.

It is noteworthy that oxidative stress, resulting from the proliferation of free radicals, has essentially been implicated in the pathophysiology of AD [42, 43]. However, a list of natural plants rich in bioactive constituents have been reported to exhibit antioxidative properties either by chelating, scavenging or inhibiting the initial of ROS [9, 44]. Previous studies have similarly established a compelling correlation between the ABTS decolorization assay and the antioxidant capability of medicinal plants [45, 46]. This decolorization is based on the ability of any compound to donate electrons and cause the inhibition of ABTS radical cations generated through chemical reduction [47]. Nonetheless, the current investigation reveals that AETt exhibited a remarkable reduction in ABT radicals (Fig. 1), suggesting the capability of the extract to donate electrons to water-soluble and chemically stable ABT, ultimately causing its reduction [48].

The cholinergic dysfunction, marked by exacerbated cholinesterase activities (AChE and BChE), is well recognized in Alzheimer's disease (AD), the most prevalent cause of dementia. [49]. Cholinesterase play a crucial role in the hydrolysis and depletion of ACh, a neurotransmitter pivotal for cognitive and mental functions of the brain [2, 50]. Consequently, the inhibition of AChE and BChE has been documented as a significant strategy for managing AD, thus rendering it a pertinent target for the development of medications aimed at addressing AD [51]. Furthermore, recent studies have underscored the significance of medicinal plants in addressing AD as a result of the existence of bioactive secondary metabolites that have been identified as potential inhibitors of AChE and BChE [40, 52]. In our study (Fig. 2), it is evident that AETt exhibited a clear concentration-dependent inhibition of AChE and BChE activities, an effect that could probably be linked presence of compounds (Table S1 & Fig. S1) with the ability to donate H-atom (H^+^), thus causing the inhibition of these hydrolyzing proteins at their catalytic sites [41]. As a result, it could be suggested that AETt holds the potential for neuroprotective effects valuable in the therapeutic management of AD.

### Molecular docking/molecular dynamics simulation

Over a span exceeding three decades, computer-aided methods for drug discovery and design have made substantial contributions to the development of essential bioactive therapeutic molecules, while also anticipating potential derivatives that could enhance their efficacy [53]. Currently, the application of in silico techniques, such as chemoinformatics, molecular modeling, and artificial intelligence (AI), has experienced substantial growth, particularly given the crucial role that understanding the molecular foundations of drug interactions plays in drug discovery [54].

From our findings, in order to screen and find acceptable hits that best fit into the most favorable binding mode having the right geometry and complementarity, four prominent leads from AETt with the lowest SP, XPG and MMGBSA scores (Tables 3 and 4), were docked against human AChE (PDB ID: 4EY7) and BChE (PDB ID: 6QAE) proteins. However, the SP scoring algorithm involves the evaluation of van der Waals, electrostatic, and hydrogen bonding interactions between proteins and the hits [55]. This method is less precise when compared to XPG and the computationally intensive MMGBSA scoring technique [56]. The MMGBSA scoring method combines molecular mechanics and continuum solvation models to calculate energy contributions, resulting in a more accurate determination of binding affinity. More so, research suggests that reduced SP and XPG values are indicative of robust interactions and compatibility between individual ligands and the specific protein target being studied [57]. Remarkably, within this array of compounds, rutin and quercetin exhibited noteworthy binding affinity energies [58]. These computed binding energies underscore the potential for enhanced binding capability of these two flavonoids with the respective target proteins, surpassing the effectiveness of the standard drugs lycodoline and scopoletin [59].

**Table 4:** ADMET/ADME properties of bioctive lead compounds from the aqueous extract of *Talinum triangulare* leave (AETt)

Compounds	Absorption				Distribution			Metabolism		Excretion	Toxicity		
	Caco-2-Perm	Pgp-inhibitor	Pgp-substrate	HIA	PPB (%)	VD	BBB-Penetration	Inhibitor	Substrate	CL	hERG-Blockers	H-HT	Carcin
Rutin	-6.47	0.005	0.997	0.876	87.1	0.701	0.041	CYP3A4, CYP1A2, CYP2C19, CYP2D6, CYP2C9	CYP2C9, CYP1A2, CYP2C1,9, CYP3A4, CYP2D6,	1.50	0.227	0.083	0.055
Quercetin	-5.20	< 0.100	< 0.100	< 0.100	95.5	0.580	< 0.100	CYP1A2, CYP2C19, CYP2C9, CYP2D6, CYP3A4	CYP1A2, CYP2C9, CYP2C19, CYP2D6, CYP3A4	8.28	< 0.100	< 0.100	< 0.100

Key: *Caco-2* Human colon adenocarcinoma cell lines, *Pgp* P-glycoprotein, inhibitor or substrate, *HIA* human intestinal absorption, *PPB* Plasma protein binding, *VD* Volume of distribution, *BBB-Penetration* blood–brain barrier penetration, *CYP* Cytochrome, *CL* Clearance, *hERG* Blockers, *H-HT* Human hepatotoxicity, *Carcin* Carcinogencity

Furthermore, the complexes formed by the hits with cholinesterases exhibit notable high binding affinities, including parameters such as VDWAALS, EEL, EGB, ESURF, ΔG gas, ΔG solv, and ΔG total, as revealed by MMGBSA analysis (Fig. 3 a&b). These high binding affinities substantiate the presence of robust and stable interactions between the ligands and respective cholinesterases, which could ultimately translate to the inhibition of their enzymatic activities [60]. This finding supports the viability of these complexes as potential target in drug development [61]. Moreover, these distinct properties can potentially be attributed to the diverse hydrogen and hydrophobic bonding interactions exhibited by the hits with different amino acid residues of the protein targets (illustrated in Figs. 4 (a&b) and 5 (a&b)).This observation is consistent with the findings of Chowdhury et al. [62]. Both the hydrogen bonds and hydrophobic interactions assume pivotal roles in drug discovery and design [63]. Hydrogen bonds are indispensable, not only for facilitating drug-receptor binding but also for influencing a molecule's properties like solubility, distribution, and permeability [64]. Similarly, hydrophobic interactions are vital in determining the binding affinity and selectivity of small molecular drugs for their target, playing a significant role in biomolecular recognition [65]. Additionally, the hydrogen bond interactions and hydrophobicity of the hits derived from AETt could possibly contribute to the favorable pharmacological ADMET parameters observed for rutin and quercetin, as presented in Table 4 [66]. Research has indicated that compounds possessing acceptable ADMET profiles are more likely to demonstrate effectiveness and safety [67]. Understanding and optimizing these ADMET properties are critical in the drug development process and the kinetics of drug exposure to tissues [68]. The ADMET characteristics of rutin and quercetin demonstrate that these compounds possess drug-like attributes that are safe and non-toxic. This probably suggests their potential usefulness in AD drug development.

Radius of gyration (Rg) is another invaluable parameter used to evaluate the conformational properties of AChE (PDB ID: 4EY7) and BChE (PDB ID: 6QAE) complexation with rutin and quercetin in molecular dynamics simulation. A molecular dynamics simulation provides insights into the conformational dynamics and structural stability of the protein–ligand complex by keeping track of changes in the Rg [69]. The Rg value has been used as a measure of the compactness and stability of protein with ligand [70]. It also quantifies the distribution of the atoms in the complex relative to the centre mass. A more broad or unfolded structure is indicated by an increase in the Rg value than a more compact or folded structure [71]. As seen in our study (Fig. 6), 4EY7-rutin and 6QAE-quercetin indicated moderate Rg value compared to the apoproteins used. However, 6QAE-quercetin showed a more compacted or folded structures. Similarly, a more common method for assessing the flexibility and local dynamics of a protein–ligand complex during a molecular dynamics simulation is root mean square fluctuation (RMSF) analysis [72]. RMSF provides details on the parts of the complex that are more rigid or suffer from large fluctuations [73]. As indicated in Fig. 7 (a&b), AChE-rutin (4EY7-rutin) complex had the highest RMSF (Ǻ) value compared to other complexes. Lower RMSF values indicate rigid or stable regions, while higher RMSF values suggest regions with greater flexibility or larger structural fluctuations [74]. However, the complexes maintained their stability throughout the simulation, as indicated by the average RMSD values (Fig. 8), which implies profound protein–ligand interactions. The RMSD describes the measure of the changes in the conformation of a given structure over time and offers details on the complexes' long-term stability and convergence.

**Fig. 11:**
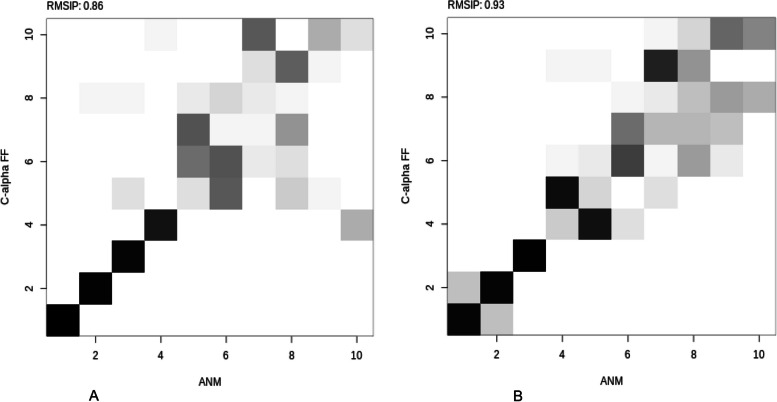
Mode similarity analyses between anisotropic network model and C-alpha force fields using RMSIP for **A** acetylcholinesterase-rutin complex and **B** butyrylcholinesterase-quercetin complex. ANM, anisotropic network model

The protein's structural changes caused by rutin and quercetin dynamics binding were also verified using principal component analysis (PCA) that revealed the overall motion of the molecular dynamics trajectories [75]. As seen in Figs. 9 and 10, the top five eigenvectors in the human AChE and BChE complexes showed dominating movements with eigenvalues between 22.1–47.1% and 14–43.9%, respectively. In general, all clusters showed conformational changes with the blue region presenting the most significant movements, the white region representing intermediate movements, and the red zone showing the least flexible motions, according to the PCA. For the AChE-rutin (Fig. 9), PC1 had the highest variance, followed by PC2 and PC3. This suggests that PC1 captured the most significant structural changes induced by the ligand binding, while PC2 and PC3 captured more subtle changes in the orientation and conformation of different functional groups [76]. Similarly, for the BChE-quercetin (Fig. 10), PC1 had a lower variance, while PC2 and PC3 had higher variances. This implies that the ligand induced more subtle structural changes in the protein as captured by PC2 and PC3, while PC1 revealed the most significant structural changes according to the report of Laerge and Yonetani [77]. Additionally, investigation of mode similarity analyses between anisotropic network model and C-alpha force fields were revealed using root mean square inner product (RMSIP) in 4EY7-rutin and 6QAE-quercetin. In the plots, the protein residues are displayed as pixels in a heatmap, and the colour of each pixel indicates the RMSIP value for an individual protein residue (Fig. 11 a&b). Darker colour typically corresponds to lower RMSIP values, and this denotes lower similarity between the two force fields' predicted normal modes, whereas lighter colours correspond to higher RMSIP values and similarly denote greater similarity.

## Conclusion

Our study investigated the antioxidant (ABTS cation radicals inhibition) and enzyme (cholinesterase and butyrylcholinesterase) inhibitory activities of the aqueous extract of *Talinum triangulare* leave (AETt). Also, molecular docking and simulation studies were performed to explore the interaction of previously identified bioactive compounds in AETt with human cholinesterase enzymes. The results however, indicated noticeable ABTS radical and cholinesterase inhibitory activities as exhibited by AETt. More so, molecular docking simulations identified rutin and quercetin from AETt as promising drug candidates, demonstrating strong binding affinities with human cholinesterase enzymes. Also, further computational analyses indicated structural stability, compactness, and stable interactions of the acetylcholinesterase-rutin and butyrylcholinesterase-quercetin complexes. Overall, our study could offer valuable insights into the radical scavenging and cholinesterase inhibitory potential of AETt, thereby providing a premise for drug development useful in the management of Alzheimer’s disease.

### Supplementary Information

**Supplementary Material 1**.

## Data Availability

Proteins analyzed such as human acetylcholinesterase (AChE) (PDB ID: 4EY7) and butyrylcholinesterase (BChE) (PDB ID: 6QAE) were obtained from protein data bank (https://www.rcsb.org/).

## Abbreviations

µg: Microgramme
Abs: Absorbance
ABTS: 2,2- Azinobis (3-ethyl-benzothiazoline-6-sulfonic acid)
ACh: Acetylcholine
AChE: Acetylcholinesterase
AD: Alzheimer’s disease
ADME: Administration, distribution, metabolism and excretion
ADMET: Administration, distribution, metabolism, excretion and toxicity
AETt: Aqueous extract of *Talinum triangulare* leave
ARG: Arginine
ASP: Aspartic acid
BCh: Butyrylcholine
BChE: Butyrylcholinesterase
BHT: Butylated hydroxytoluene
BHT: Butylated hydroxytoluene
ChE: Cholinesterases
DTNB: 5,5’-Dithiobis (2-nitrobenzoic acid)
EC: Enzyme code
EEL: Electrostatic
EGB: Generalized born electrostatic
ESURF: Nonpolar solvation
GB/SA: Generalized-Born/surface area
GLN: Glutamine
GLU: Glutamic acid
GLY: Glycine
HIS: Histidine
HPLC–DAD: High performance liquid chromatography coupled with diode-array detection
IC_50_: Concentration that can cause 50% inhibition
LEU: Leucine
m: Molarity
MDS: Molecular dynamics simulation
ml: Milliliter
mM: Millimole
MM-GB/SA: Molecular mechanics/generalized born surface area
PBS: Phosphate buffer Saline
PC: Principal component
PCA: Principal component analysis
PHE: Phenylalanine
Rg: Radius of gyration
RMSF: Root mean square fluctuation
RMSIP: Root mean square inner product
ROS: Reactive oxygen specie
RT: Room temperature
SER: Serine
SP: Standard precision
TRP: Tryptophan
*Tt*: *Talinum triangulare*
TYR: Tyrosine
UV: Ultraviolet
VAL: Valine
VDWAALS: Van der Waals
XP: High precision
ΔG: Free energy of binding
ΔG solv: Free energy of solvation
ΔG total: Total free energy of binding
