# Supplementary material for: Insight into antioxidant-like activity and computational exploration of identified bioactive compounds in Talinum triangulare (Jacq.) aqueous extract as potential cholinesterase inhibitors
Source: BMC Complement Med Ther. 2024 Mar 28;24:134. doi: 10.1186/s12906-024-04424-2 (PMC10976795; doi:10.1186/s12906-024-04424-2)
Supplement: Supplementary file 1 — Supplementary Material 1. [file 12906_2024_4424_MOESM1_ESM.docx]

Supplementary Table 1 (Table S1): HPLC analyses of the aqeuous extract of *Talinum triangulare* leave (AETt)

| Compounds AETt LOD LOQ  mg/g μg/mL μg/mL  Chlorogenic 1.43 ± 0.02 0.027 0.089  Caffeic acid 1.45± 0.02 0.009 0.030  Rutin 0.37± 0.01 0.011 0.036  Quercetin 3.68± 0.03 0.016 0.053  Kaempferol 0.00± 0.00 0.025 0.082  Luteolin 0.97± 0.04 0.010 0.033  Apigenin 2.04± 0.03 0.019 0.062 |
| --- |
| The values for the various samples were expressed as mean ± SD of triplicate determinations (n=3) [43]. |


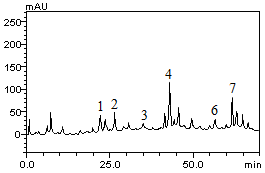


Figure S1: High performance liquid chromatographic analysis of aqueous extract of *Talinum triangulare* leave [43].

Note: Peak 1, chlorogenic acid; peak 2, caffeic acid; peak 3, rutin; peak 4, quercetin; peak 5 kaempferol; peak 6, luteolin; and peak 7, apigenin.
